# Sarm1 induction and accompanying inflammatory response mediates age-dependent susceptibility to rotenone-induced neurotoxicity

**DOI:** 10.1038/s41420-018-0119-5

**Published:** 2018-12-11

**Authors:** Malinki Sur, Puja Dey, Ankita Sarkar, Sudipta Bar, Dipanjana Banerjee, Swati Bhat, Piyali Mukherjee

**Affiliations:** 1 Department of Life Sciences, Presidency University, 86/1, College Street, Kolkata, 700073 West Bengal India; 2 Department of Biological Sciences, IISER Kolkata, Mohanpur, 741246 West Bengal India

## Abstract

Aging is a complex biological process and environmental risk factors like pesticide exposure have been implicated in the increased incidence of age-related neurodegenerative diseases like Parkinson’s disease (PD) but the etiology remains unknown. There is also lack of a proper animal model system to study the progressive effect of these environmental toxins on age-associated neurodegeneration. In this study, we established a drosophila model of aging to study the age-dependent vulnerability to the environmental toxin rotenone that has been implicated in sporadic cases of PD. We demonstrate that age plays a determining role in the increased susceptibility to chronic rotenone exposure that is accompanied by severe locomotor deficits, decreased lifespan and loss of dopaminergic (DA) neurons. Chronic low dose exposure to rotenone results in the rapid induction of the neurodegenerative molecule SARM1/dSarm. Further, the age-dependent dSarm induction is accompanied by a heightened inflammatory response (increased expression of *Eiger* and *Relish*) that is independent of reactive oxygen species (ROS) generation in the observed rotenone-induced neurotoxicity. dSarm induction and subsequent locomotor deficits is reversed in the presence of the anti-inflammatory molecule resveratrol. Thus, dSarm and heightened inflammatory responses may play a crucial role in age-dependent vulnerability to the pesticide rotenone thus making it an attractive target to help develop cost-effective therapeutic strategies to prevent ongoing dopaminergic neuronal loss as seen in PD.

## Introduction

Neurodegenerative diseases affect the central nervous system (CNS) causing progressive loss of neuronal structure and function^1,2^. An important subset of these disease concerns age-related neurological disorders like Alzheimer’s disease (AD) and Parkinson’s disease (PD) which are debilitating and incurable and is estimated to affect about 30 million people worldwide^3^. Gene–environment interaction has been implicated in the increased incidence of these disease and environmental stressors like pesticide exposure may accelerate age-related neuronal loss. However, there is a lack of a proper model system to study the progressive nature and the molecular mechanism of this neuronal loss that is sporadic in nature. Several mouse models have been developed for the study of PD but the variation between inbred strains and the striking difference in their response to experimental interventions from humans poses serious limitations in their use for such a study.

Rotenone is a naturally occurring insecticide that is highly lipophilic and readily crosses the blood–brain barrier and has been implicated in dopaminergic neuronal loss as often seen in PD^4–6^. Rotenone treatment in rats mimic many of the PD like symptoms and associated histolopathological features and is a widely used model for the study of the underlying mechanism of sporadic PD. Rotenone is a mitochondrial complex I inhibitor and a few studies have implicated that rotenone causes elevated levels of reactive oxygen species (ROS) but how this increased ROS may subsequently lead to dopaminergic neuronal loss is not clearly understood. Studies in Drosophila model have shown that chronic rotenone exposure of flies could result in dopaminergic neuronal loss accompanied by locomotor deficits and increased generation of ROS^7,8^. However, these studies fail to explain the fact that replenishing dopamine relieve initial symptoms but cannot establish a long-lasting cure to the motor deficits observed in PD which is probably associated with a rapid neuronal loss at later stage of the disease. Further, these studies examined the effect of chronic rotenone exposure in younger flies (7-days following exposure) but not the age-dependent vulnerability to low-level chronic exposure to such environmental toxins. Thus, an in-depth analysis of the mechanisms underlying the loss of dopaminergic neurons and how age acts as a trigger for the increased susceptibility to loss of these neurons is required to explain this phenomenon.

Axonal loss is an early pathology of aging where axons from the synaptic region gradually degenerate to the cell body in a ‘dying back’ phenomenon^9,10^ although the molecular mechanisms that cause/regulate axonal retraction during aging is not known. A recent study pinpointed a protein named dSarm/Sarm1 in *Drosophila melanogaster* that was shown to play a pivotal role in programmed axonal degeneration^11^ and depletion of NAD^+^ and subsequent energy deficit in the axons played an important role in this process^12,13^. Previous studies have also implicated the importance of Sarm1 in neuronal death associated with cerebral ischemia or stroke^14^. Another study in a neuronal model of virus infection demonstrated that localization of Sarm1 at the mitochondria plays a key role in mitochondrial dysfunction leading to excess ROS generation and ultimately neuronal death^15^. Sarm1 is one of the first identified human TIR containing protein that was shown to possess NADase activity^13^ with the subsequent identification of such a mechanism in lower organisms as well^16^. Interestingly, it has been recently shown that Sarm1 mediate neuronal death induced by rotenone in primary neuronal cultures^12^. Collectively, these studies point toward the important role of Sarm1 in mediating neuronal death in various models of neurodegeneration^17,18^ although its role in age-associated neurodegeneration remains elusive so far.

Taking cues from such studies we have shown here that chronic rotenone exposure leads to locomotor deficits and decreased survival in Drosophila in an age-dependent manner. This is accompanied by increased induction of *Ect 4* or *dSarm* (Drosophila homolog of Sarm1) along with the inflammatory molecule *Eiger* (Drosophila homolog of TNFα). The increased susceptibility to rotenone-induced toxicity increases with age that correlates with an early induction of *dSarm* along with the inflammatory response that is independent of ROS generation contrary to earlier studies. Rotenone has also been previously shown to induce an inflammatory response through microglial activation^19,20^ but these studies failed to correlate this heightened inflammatory response with the dopaminergic neuronal loss or the increased motor deficits as often seen in PD. However, analysis of other immune response genes in our Drosophila model demonstrated the rotenone-induced inflammatory response to be specific to *Eiger* induction that is accompanied by JAK/STAT-mediated sterile inflammatory pathway. Interestingly, rotenone-induced locomotor deficits and increased mortality are significantly reversed in flies fed with the anti-inflammatory molecule resveratrol which also reversed the early induction of *dSarm* along with the accompanying motor deficits. This study proposes for the first time that Sarm1 could be one of the key molecule for increased susceptibility to rotenone-induced neurotoxicity in the aging flies and may serve as a downstream effector of inflammatory responses, the implications of which in age-associated neurodegeneration needs to be explored further.

## Results

### Rotenone exposure causes reduced lifespan and progressive locomotor deficits in w^1118^ strain of Drosophila in an age-dependent manner

**Fig. 1:**
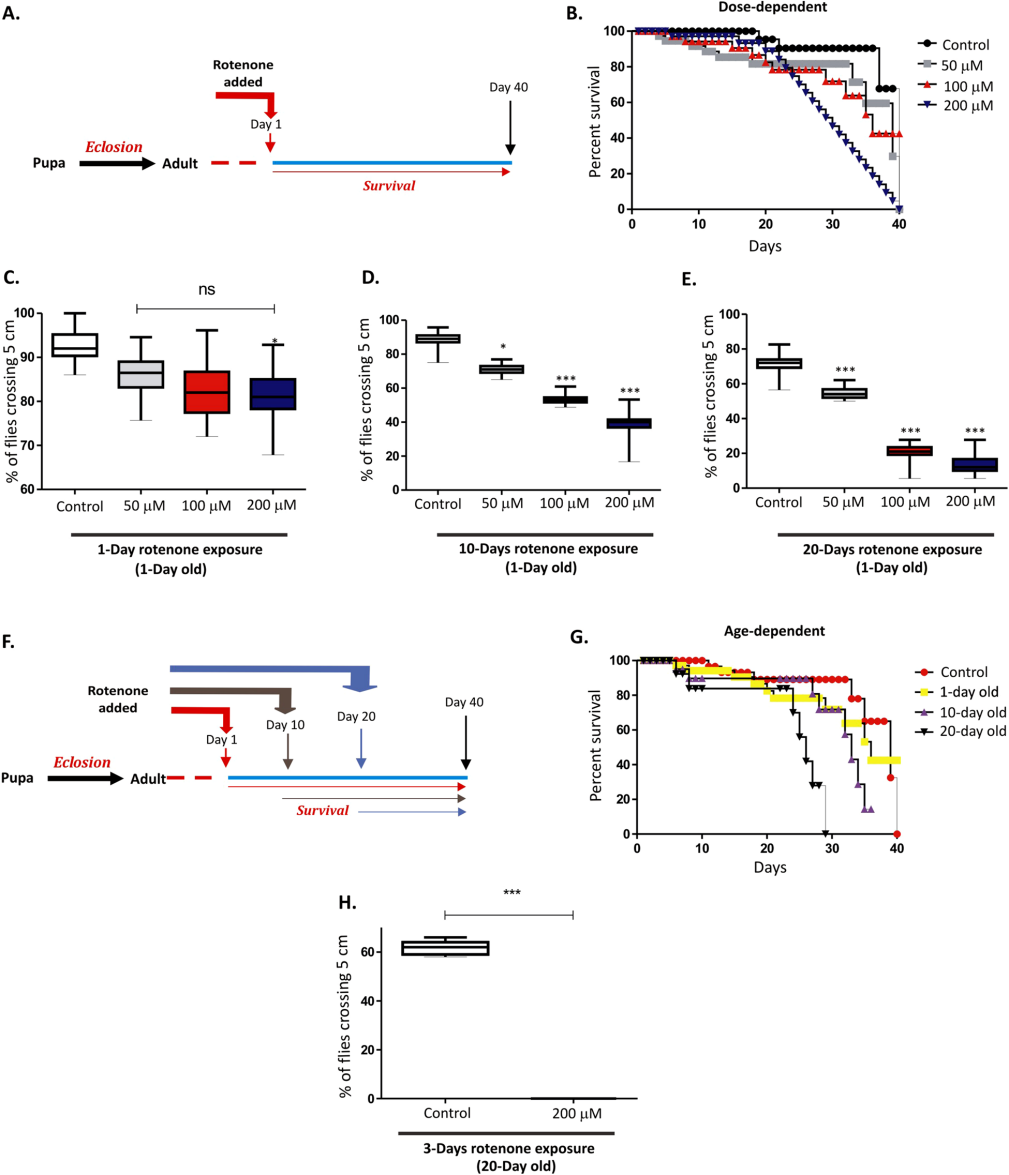
Exposure of w^1118^ flies to rotenone-induces shortened lifespan and severe locomotor deficits in a dose- and age-dependent manner. **a** Schematic representation of rotenone treatment in the 1-day old flies for survival curve assay. **b** Survival curve of 1-day old flies exposed to varying concentration of rotenone (50 , 100 , and 200 μM). Fly viability was scored over a period of 40-days using a minimum of 100 flies per treatment. The statistical significance was calculated as log-rank using Mantel–Cox test. **c**–**e** Negative geotaxis assay of 1-day old flies exposed to rotenone at concentrations indicated above for 1-day (**c**), 10-days (**d**) and 20-days (**e**). **f** Schematic representation of rotenone treatment in the 1-day, 10-day, and 20-day old flies for survival curve assay. **g** Age-dependent survival of flies exposed to 100 µM of rotenone at 1-day, 10-day, and 20-day following eclosion. Fly viability was scored up to 40-days, post-eclosion using a minimum of 100 flies per treatment. **h** Negative geotaxis assay of 20-day old flies exposed to 200 μM rotenone for 3-days. *p *<* 0.05, **p < 0.01, and ***p < 0.001 compared to control flies

To study the effect of the pesticide rotenone on Drosophila survival, w^1118^ flies were exposed to increasing doses of rotenone 1-day post-eclosion and survival of the flies were followed up to 40-days as represented in Fig. 1a. As shown in Fig. 1b the flies were susceptible to increasing concentration of rotenone (50, 100, and 200 μM) that was accompanied by increased motor deficits as shown by their decreased climbing ability in a negative geotaxis assay at 1-day (Fig. 1c), 10-days (Fig. 1d) and 20-days (Fig. 1e) post-treatment. One-way ANOVA indicates that climbing ability in 200 μM rotenone-exposed adult w^1118^ flies was significantly (*P* < 0.001) less than that of untreated flies after 1-day, 10-days, and 20-days rotenone exposure.

To determine the age-related susceptibility to environmental toxins, flies were exposed to the sub-lethal concentration (100 μM) of rotenone at day-1, day-10 and day-20 following eclosion (Fig. 1f). Interestingly, age played a significant role in the increased susceptibility of these flies to rotenone exposure. In flies exposed to rotenone at 20-days following eclosion (older flies), the death rate was significantly increased (*P* < 0.001) compared to the younger flies (Fig. 1g) which was accompanied by severe motor deficits (Fig. 1h).

### Age-dependent susceptibility to rotenone is accompanied by increased *dSarm* expression

**Fig. 2:**
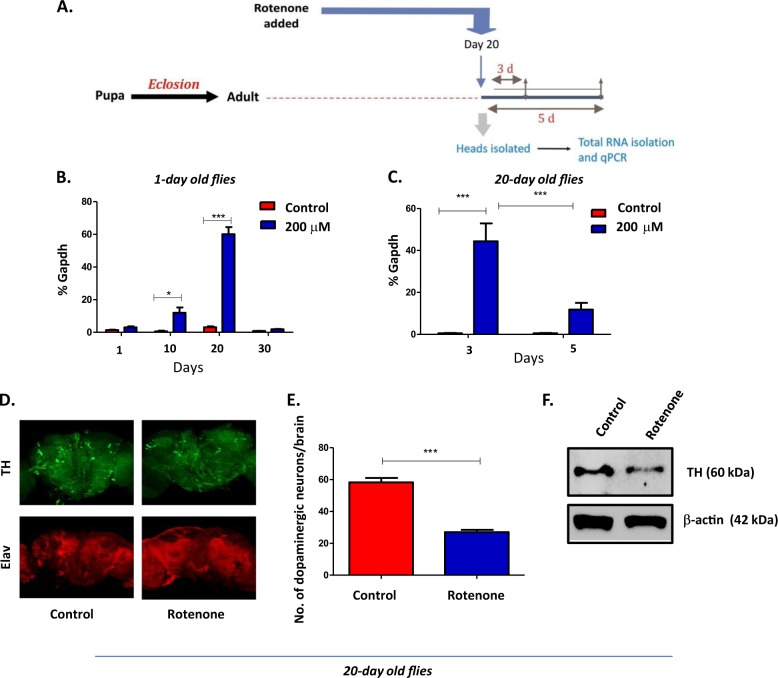
Exposure of w^1118^ flies to rotenone causes rapid loss of dopaminergic neurons in aged flies accompanied by increased *dSarm* expression. **a** Schematic representation of experimental setup for rotenone treatment in the aged (20-days old) flies. **b**, **c** Expression of *dSarm* in young flies (1-day old) exposed to 200 μM of rotenone for 1-day, 10-days, 20-days, and 30-days (**b**) and aged flies (20-days old) to 200 μM of rotenone for 3-days and 5-days post-treatment (*n* = 5) (**c**). **d** Multifocal confocal images of dopaminergic neurons following tyrosine hydroxylase (TH) immunostaining (green) and Elav immunostaining (red) in the brains of flies exposed to 200 μM rotenone at 20-days post-eclosion for 3-days (right-hand panel). Results were compared to age-matched rotenone untreated flies (left-hand panel). Scale bars = 200 μm. **e** Bar graph represents the number of dopaminergic neuron cell bodies in the control and rotenone (200 μM) treated fly brains (*n* = 3). **f** Representative western blot analysis of the dopaminergic neuronal marker tyrosine hydroxylase in 20-day old flies exposed to 200 μM rotenone and analyzed at day-3 post-exposure. β-actin served as a loading control and data was compared with age-matched untreated control flies (*n* = 3). *p < 0.05, **p < 0.01 and ***p < 0.001 compared to control flies

Clearly our data indicate that age is a risk factor for increased susceptibility to rotenone-induced neurotoxicity. In an attempt to identify the molecular mechanism for this increased susceptibility we analyzed the expression of one of the neurodegenerative molecule *dSarm* (Drosophila homolog of Sarm1) that has been implicated in injury-induced axonal loss in flies^11^ in the young (1-day old) and aged (20-days old) flies as represented schematically in Figs 1a and 2a. An early induction of *dSarm* was observed in the brain of young flies as early as 10-days post-exposure, which peaked at 20-days (greater then 3-fold increase compared to 1-day old flies) followed by a steep decrease at 30-days post-exposure (Fig. 2b). To further understand the age-dependent susceptibility to rotenone, aged flies (20-day old) were exposed to 200 μM rotenone and brains were collected at 3-days and 5-days post-exposure. These flies were highly susceptible to rotenone exposure and did not survive beyond 10-days post-exposure. As compared to the younger flies there was a significant increase in *dSarm* expression (Fig. 2c) as early as 3-days post-exposure in these aged flies followed by a rapid decrease at 5-days which correlated with the increased age-related susceptibility to rotenone.

It has been previously shown in flies that rotenone treatment induces dopaminergic neuronal loss in younger flies (7-day old) exposed to rotenone^17^. In agreement with our earlier data of age-related increased susceptibility to rotenone, we observed a rapid and significant loss of dopaminergic neurons from all the dopaminergic neuronal clusters indicated by tyrosine hydroxylase (TH) staining of whole brains in the 20-day old flies exposed to rotenone for 3-days as compared to age-matched control flies (Fig. 2d, e). We also observed a significant reduction of TH protein levels in the 20-day old flies as early as 3-days post-exposure with 200 μM of rotenone as compared to their age-matched controls (Fig. 2f).

### An early induction of dSarm was found to be necessary and sufficient for locomotor deficits following exposure to rotenone

**Fig. 3:**
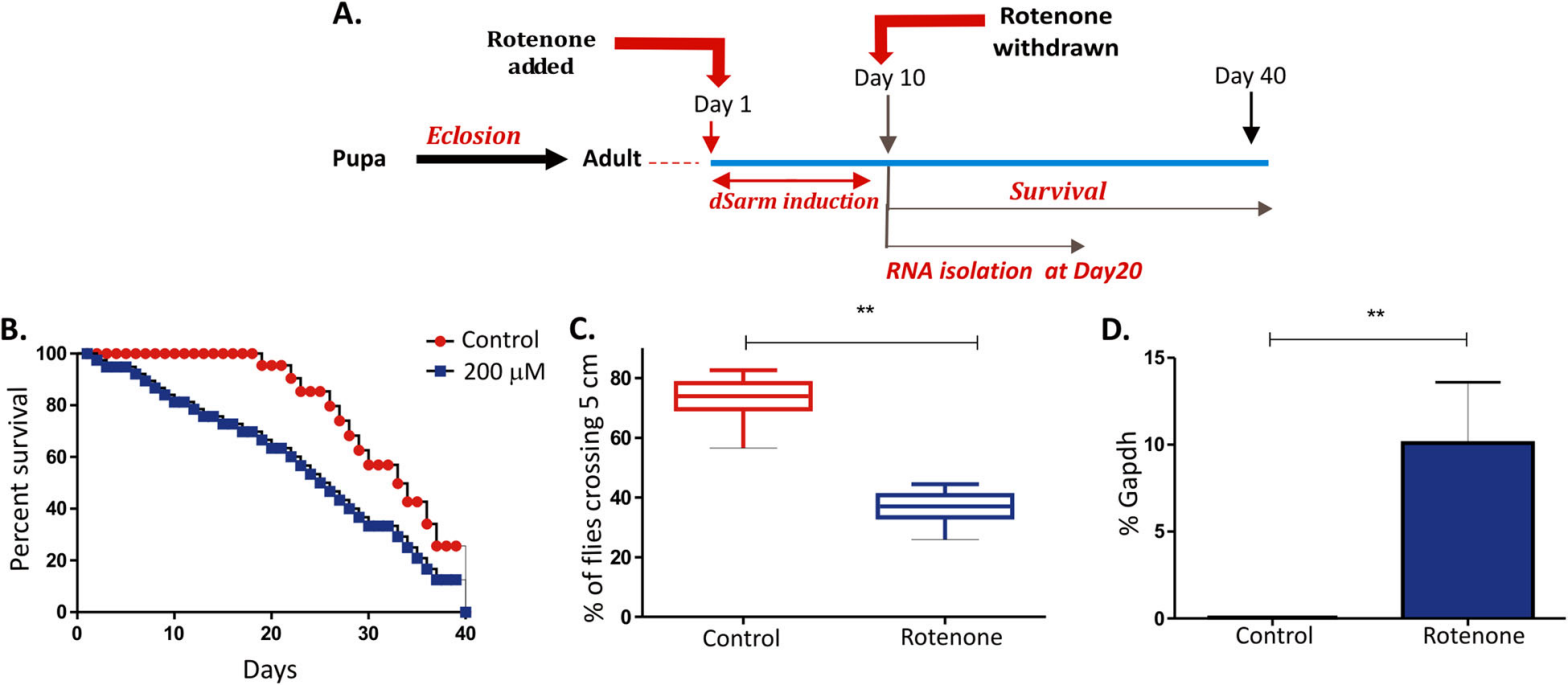
Early induction of *dSarm* is necessary and sufficient for rotenone-induced neurotoxicity. **a** Schematic representation of rotenone treatment of 1-day old flies for 10-days followed by withdrawal and further experiments were performed at the time points indicated. **b** Survival curve of control adult flies and flies exposed for 10-days to 200 μM of rotenone and subsequent transfer of these flies to normal media. Fly viability was scored over a period of 40-days using a minimum of 100 flies per treatment. **c** Negative geotaxis assay of adult flies exposed to rotenone as indicated above for 10-days and experiment performed at day-20 post-exposure. **d** *dSarm* expression was analyzed at day 20 of flies treated above and results compared to age-matched untreated control flies. *p < 0.05, **p < 0.01 and ***p < 0.001 compared to control flies

To understand whether the early induction of dSarm as observed in Fig. 2b was sufficient to initiate rotenone-induced neurotoxicity, flies were exposed to 200 μM of rotenone for 10-days following which they were grown in media without rotenone (Fig. 3a). An early exposure to rotenone in the younger flies for 10-days and subsequent removal and growth of these flies in rotenone-free media continued to induce loss of survival (Fig. 3b) and locomotor deficits (Fig. 3c). A paired student t-test indicated significant (*P* < 0.01) upregulation of *dSarm* expression following early rotenone exposure (Fig. 3d) in these flies. Taken together, these data suggest that early Sarm1 induction may act like a switch to trigger the loss of dopaminergic neurons leading to rotenone-induced motor deficits in flies.

### Rotenone-induced locomotor deficits are not regulated by increased ROS generation

**Fig. 4:**
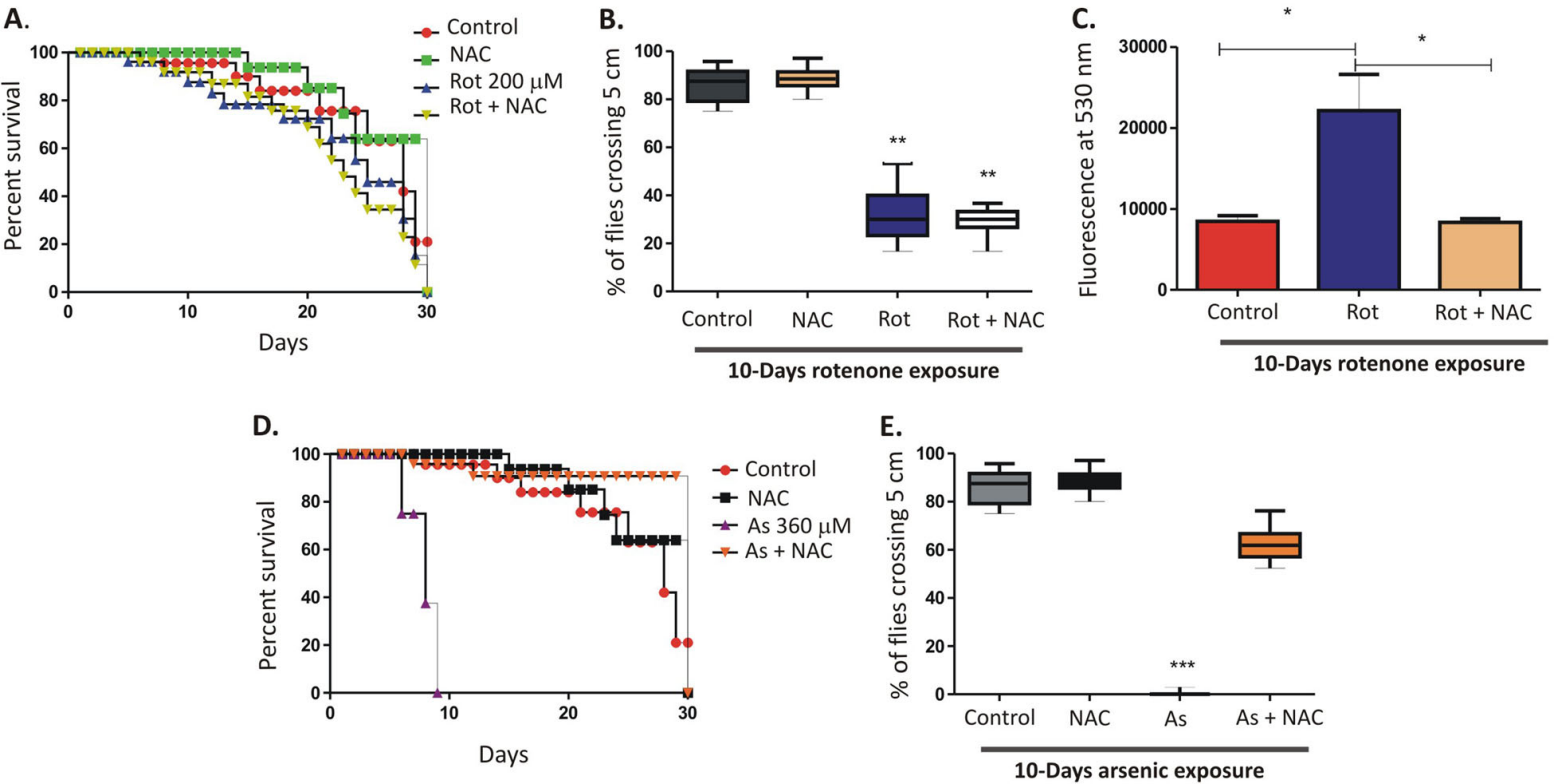
Rotenone-induced locomotor deficits are not regulated by increased ROS generation. **a** Survival curve of control adult flies and flies exposed to 200 μM of rotenone in the presence or absence of the ROS scavenger N-acetyl-cysteine or NAC (1 mg/ml). Fly viability was scored over a period of 30-days, using a minimum of 100 flies per treatment. **b** Negative geotaxis assay of the flies at 10-days post-exposure. **c** ROS generation assay in 1-day old flies exposed to 200 μM rotenone for 10-days in the presence or absence of NAC. **d** Survival curve of control adult flies and flies exposed to 360 μM of Arsenic (As) along with NAC (1 mg/ml). Fly viability was scored over a period of 30-days, using a minimum of 100 flies per treatment. **e** Negative geotaxis assay of the flies exposed to As in the presence or absence of NAC at 10-days post-exposure. *p < 0.05, **p < 0.01 and ***p < 0.001 compared to control flies

The free radical theory of aging proposed by Harman et al. in 1950s suggests an accumulation of ROS contributes to aging^21^. However, this theory has been controversial and antioxidant therapy has been found to reverse shortened lifespan only in a few models^22^. The pesticide rotenone has been shown to induce neuronal apoptosis via ROS generation^23^. Taking cue from these observations, we exposed w^1118^ flies with rotenone along with the antioxidant N-acetylcysteine (NAC). Interestingly, NAC did not reverse rotenone-induced toxicity (Fig. 4a) as well as locomotor deficits (Fig. 4b) in these flies. However, ROS levels were moderately elevated in these flies following 10-days exposure to 200 μM rotenone which was reversed by the addition of NAC (Fig. 4c). To confirm the specific role of ROS in environmental toxin-induced neurodegeneration, flies were also exposed to sodium arsenite (As) in combination with NAC. Interestingly, a significant reversal of As induced toxicity (Fig. 4d) and locomotor deficits (Fig. 4e) were observed in the presence of NAC. A recent publication suggested Sarm1 worked upstream of ROS and played a major role in ROS induced neuronal death^24^. However, our data suggest that rotenone-induced ROS generation is not the key factor in the DA neuronal loss and *dSarm* induction may act independently of ROS generation.

### Age-dependent increased susceptibility to rotenone was accompanied by heightened inflammatory response

**Fig. 5:**
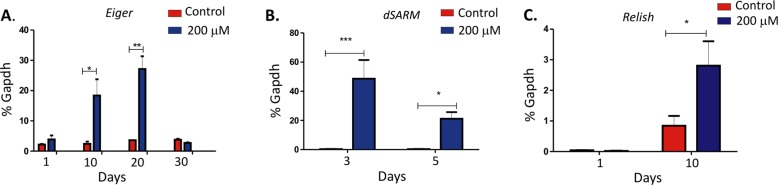
Exposure of w^1118^ flies to rotenone is accompanied by increased *Eiger* expression. **a**, **b** Eiger expression in younger (1-day old) flies exposed to 200 μM of rotenone for 1-day, 10-days, 20-days, and 30-days (**a**) and aged (20-days old) flies to 200 μM of rotenone for 3-days and 5-days post-treatment (**b**). **c** Expression of Relish in 1-day old flies exposed to 200 μM of rotenone for 1-day (left panel) and 10-days (right panel) (*n* = 5). *p < 0.05, **p < 0.01, and ***p < 0.001 compared to control flies

Sustained inflammation in the brain has been linked to several neurodegenerative diseases and results in the failure of normal resolution mechanisms of the brain^25^. In PD, production of proinflammatory cytokines IL1β and TNFα from nonneuronal population like the microglia modulates neuronal loss^25,26^. The molecule Sarm1 has been linked to modulate TNFα production to restrict viral infection^27^. Since the brain of rotenone-exposed drosophila showed increased *dSarm* expression, we also analyzed the expression of *Eiger* (TNFα homolog in Drosophila) in these flies. Significant increase (*P* < 0.01) in *Eiger* expression was observed in the brains of younger flies flies (1-day post-eclosion) exposed to 200 μM rotenone (Fig. 5a). Interestingly, *Eiger* expression followed a similar increase as observed with *dSarm* as shown in Fig. 2b followed by a decrease at 30-days post-exposure.

To correlate the age-dependent susceptibility with increased inflammatory response, 20-day old flies were exposed to rotenone and brains were collected at 3-days and 5-days post-exposure. There was a significant increase in *Eiger* expression (Fig. 5b) compared to flies similarly exposed at 1-day post-exposure. However, the inflammatory response was significantly reduced in the 5-days treated flies compared to the 3-days post-treatment that correlated with *dSarm* expression in these flies. Analysis of other immune response genes (*Upd1*, *Upd2*, *Upd3*, and *Wnt*) showed no significant increase in the flies exposed to 200 μM of rotenone in the young as well as aged flies (Fig. S1). However, our results showed that the NFκB protein Relish was upregulated in the younger flies following exposure to rotenone for 10-days (Fig. 5c) and thus may serve as an upstream activator of the Eiger pathway in the flies following rotenone treatment which needs to be investigated further.

### The anti-inflammatory molecule resveratrol reduced *dSarm* expression and rescued rotenone-induced locomotor deficits

**Fig. 6:**
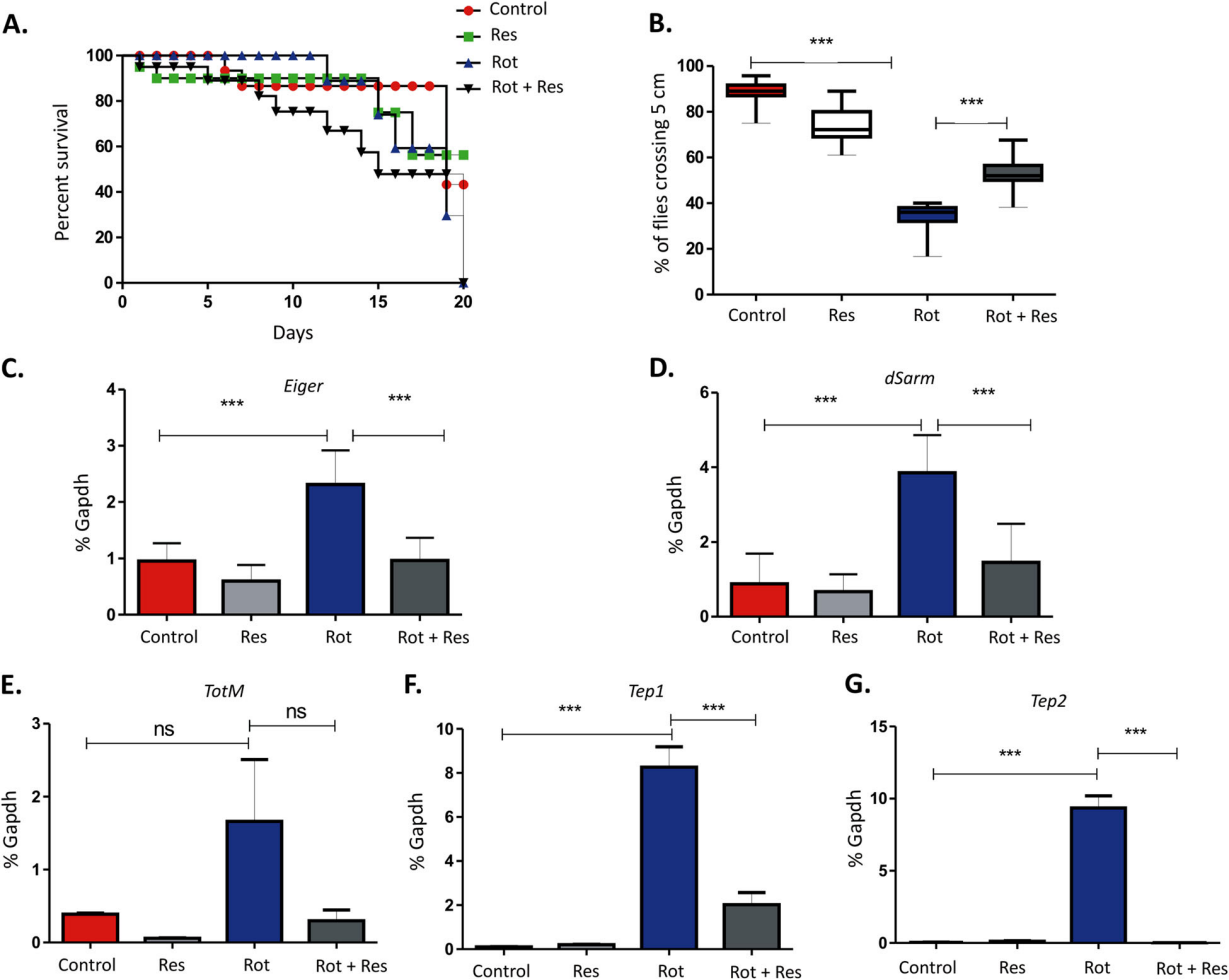
Resveratrol partially reversed early motor deficits but did not affect loss of survival. **a** Survival curve of 1-day old flies exposed to 200 μM of rotenone in the presence or absence of 1 μM of resveratrol. Fly viability was scored over a period of 20-days, using a minimum of 100 flies per treatment. **b** Negative geotaxis assay of the same samples as obtained in Fig. 7a at 10-days post-exposure. **c**, **d** Real-time PCR analysis of fly heads as treated above. Both *Ect4* (C) and *Eiger* (D) genes were analyzed in the 1-day old flies following 1-day post-exposure to rotenone (200 μM) in the presence or absence of resveratrol (1 μM) and results compared to age-matched untreated control flies. **e**–**g** Real-time PCR analysis of fly heads as treated above. *TotM* (E), *Tep1* (F), and *Tep2* (G) genes were analyzed in the 1-day old flies following 1-day post-exposure to rotenone (200 μM) in the presence or absence of resveratrol (1 μM) and results compared to age-matched untreated control flies. *p < 0.05, **p < 0.01, and ***p < 0.001 compared to control flies

The polyphenolic compound resveratrol (trans-3,4’,5-trihydroxystilbene) found naturally in grapes and nuts, has been shown to be a promising neuroprotective agent acting through the downregulation of the inflammatory responses^28^. To understand whether rotenone-induced induction of the inflammatory responses plays a role in the age-associated susceptibility, w^1118^ flies were exposed to 200 μM rotenone at 1-day post-eclosion in the presence or absence of resveratrol. Our data indicated that with increasing age, the flies were more susceptible to rotenone-induced toxicity in the presence of resveratrol (Fig. 6a). This finding correlates with the idea that mild inflammation has neuroprotective effects and complete abrogation of the inflammatory responses that occur in these flies with continuous exposure to resveratrol may have detrimental effects in the CNS. As our previous findings suggested that an early induction of *dSarm* may play an important role in the subsequent age-associated susceptibility to rotenone, we exposed the flies to 200 μM rotenone with or without resveratrol (1 μM) at 1-day post-eclosion and negative geotaxis assay were performed at 10-days following exposure. A significant reversal (*P* < 0.001) of motor deficits was observed in the presence of resveratrol in the adult flies at 10-days post-exposure where *dSarm* increases significantly as shown previously (Fig. 6b). To further investigate whether resveratrol-mediated suppression of the inflammatory responses played an important role in this process, we analysed the expression of *Eiger* in these flies as early as 1-day post-exposure. A significant reversal of the inflammatory responses as seen by the reduction in *Eiger* expression was observed in these flies in the presence of resveratrol that was accompanied by a reduction in *dSarm* expression (Fig. 6c, d respectively) that strongly suggests that the inflammatory responses may act upstream of dSarm activation in these flies.

A recent study suggested the role of DAMPs (damage-associated molecular pattern) in inducing a sterile inflammatory response through activation of the JAK-STAT pathway in Drosophila^29^. To further understand whether such sterile inflammation plays a role in rotenone-induced toxicity, we analyzed the expression of a few JAK/STAT-dependent genes in rotenone treated flies in the presence or absence of resveratrol at 10-days post-exposure to rotenone. The genes included the turandot family member (TotM) belonging to a family of immune and stress response genes and thioester-containing protein (Tep) 1 and 2. A significant increase of these genes was noted in 1-day old flies flies exposed to 200 μM of rotenone (Fig. 6e–g). Interestingly, the expression of these genes was significantly reduced in flies exposed to rotenone in the presence of resveratrol which suggest that JAK/STAT-induced sterile inflammation may play an important role in rotenone associated motor deficits in addition to the Eiger pathway.

Apart from the anti-inflammatory responses, resveratrol has been shown to induce SIRT1, a member of the NAD^+^ dependent protein deacetylase family that has an important role in aging and lifespan^30,31^. Resveratrol has also been shown to regulate AMPK responses in both SIRT1-dependent and independent manner^32^. Our results indicate that resveratrol treatment in flies exposed to rotenone elevates *Sir2* gene (drosophila homolog of mammalian Sirt1) expression levels (Fig. S2A) with no effect on *Ampk* expression (Fig. S2B), the mechanism of which needs to be further explored.

### Sarm1 is required for rotenone-induced toxicity in SH-SY5Y cells

**Fig. 7:**
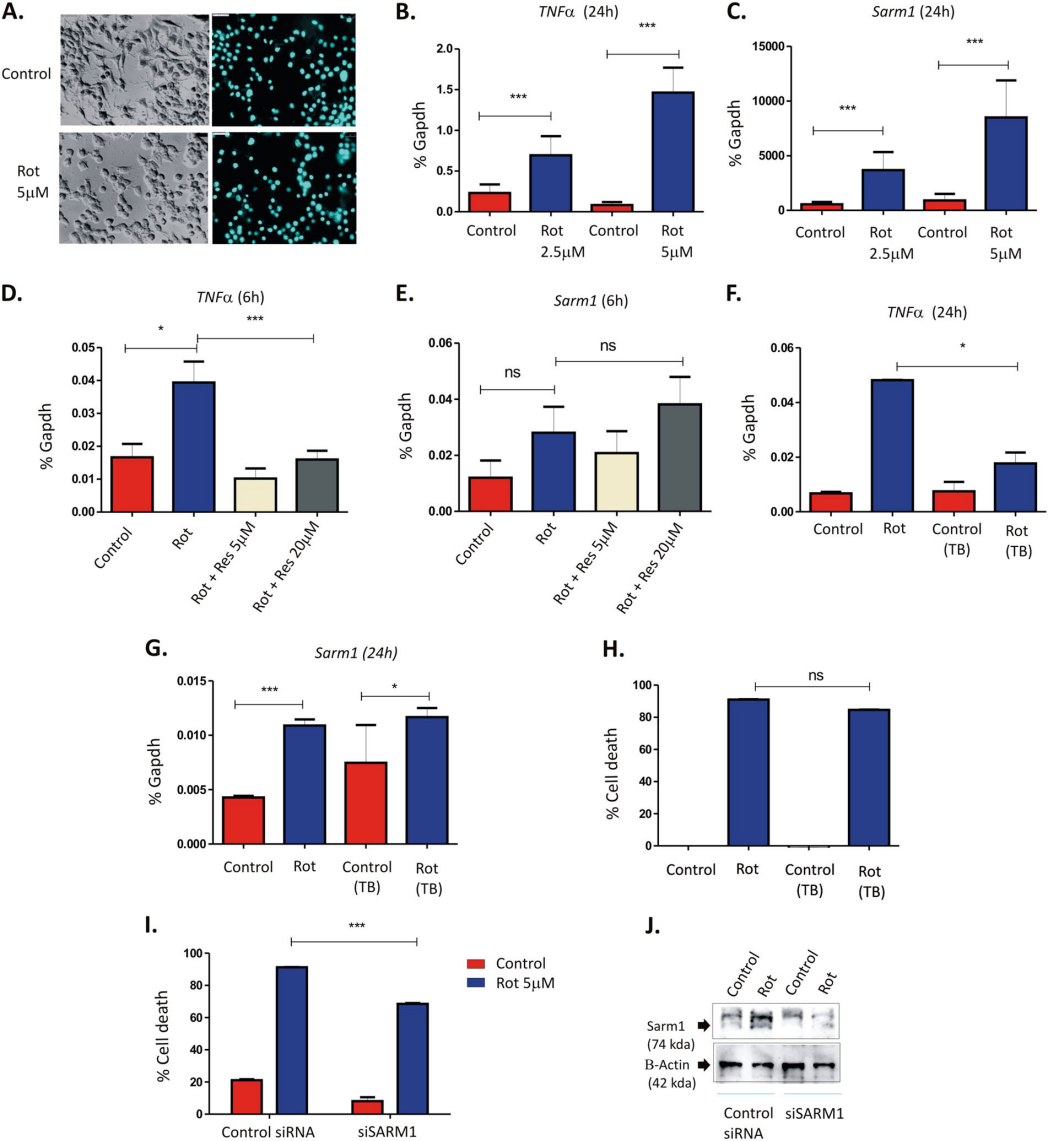
Sarm1 is required for rotenone-induced toxicity in SH-SY5Y cells. **a** Immunofluorescence analysis of SH-SY5Y cells treated with 5 μM of rotenone for 24 h (bottom panel) and compared to untreated control cells (top panel). DAPI staining of both the treated and untreated samples are shown in the right-hand panel. **b**, **c** Real-time PCR analysis of cells treated with 2.5 or 5 μM of rotenone. Both *TNFa* (**b**) and *Sarm1* (**c**) genes were analyzed following 24 h post-treatment. **d**, **e** Real-time PCR analysis of cells as treated with rotenone (5 μM) in the presence or absence of resveratrol (5 or 20 μM as indicated). Both *TNFa* (**d**) and *Sarm1* (**e**) genes were analyzed following 6 h post-treatment. **f**, **g** Real-time PCR analysis of cells treated with rotenone (5 μM) in the presence or absence of neutralizing antibody against TNFα (5 mg/ml). The expression of both *TNFa* (**f**) and *Sarm1* (**g**) were analyzed following 24 h post-treatment. **h** MTT assay of cells treated with rotenone (5 μM) in the presence or absence of neutralizing antibody against TNFα (5 mg/ml) for 24 h. **i** SH-SY5Y cells were treated with 25 nM of Sarm1 siRNA or control (nontargeting) siRNA for 24 h followed by 5 μM of rotenone treatment for 24 h. MTT assay of cells treated with rotenone in the presence or absence of siRNAs was performed. **j** Analysis of SARM1 protein levels in the whole cell extracts of cells transfected with Sarm1 or control siRNA in the presence or absence of rotenone (5 μM). β-actin was used as a loading control. Results are representative of at least three independent experiments. *p < 0.05, **p < 0.01, and ***p<0.001 compared to control flies

In order to understand whether the inflammatory response acts upstream of Sarm1 induction studies were conducted on SH-SY5Y cells which provide an excellent in vitro model for PD as it shares some characteristics of dopaminergic cells^34^. Our results indicate that rotenone (5 μM) induced neurite retraction at 24 h post-treatment (Fig. 7a) that was accompanied by increased induction of TNFα and Sarm1 (Fig. 7b, c, respectively). A significant reversal of the inflammatory response was observed in these cells at varying doses of resveratrol (5 and 20 μM) treatment (Fig. 7d) which significantly delayed rotenone-induced cell death (data not shown). Most importantly, TNFα expression preceded Sarm1 induction in these cells at 6 h post-treatment (Fig. 7e) which was not induced until 24 h post-treatment (Fig. 7c), suggesting that inflammatory responses within the neuronal cells might trigger Sarm1 activation. Blocking TNFα using neutralizing antibody against it reduced *TNF*α mRNA expression levels as expected (Fig. 7f) but *Sarm1* was moderately upregulated in these cells (Fig. 7g) following rotenone treatment for 24 h. However, blocking TNFα no significant reversal of rotenone-induced cell death was observed (Fig. 7h) indicating that the moderate increase in Sarm1 as observed in Fig. 7g was sufficient to drive rotenone-induced cell death. Interestingly, prior treatment with siRNA for Sarm1 followed by rotenone treatment significantly reversed cell death (Fig. 7i) and Sarm1 protein levels were also reduced in these cells (Fig. 7h) indicating that Sarm1 is the key mediator of the rotenone-induced neurotoxicity in these cells.

## Discussion

Why age is a risk factor for certain neurodegenerative diseases like AD or PD is a long-standing question. Environmental toxins like rotenone has been implicated in sporadic cases of PD but the mechanisms of dopaminergic neuronal loss and subsequent locomotor deficits as seen in PD remain elusive so far. In this study, we demonstrated that age plays a determining role in the increased susceptibility to rotenone-induced neurotoxicity. Young flies (1-day old) were less susceptible to a sub-lethal dose (100 μM) of rotenone as compared to the aged flies (20-days old) which correlated with the human form of the disease where age plays a crucial role in the increased incidence of these diseases.

Neuroinflammatory responses have been shown to play an important role in several psychiatric disorders but its role in age-associated neurodegeneration is emerging. To understand how inflammation might play a role in the increased susceptibility to rotenone we analyzed the expression of the inflammatory molecule *Eiger* following rotenone exposure in the 1-day old flies. We demonstrate for the first time that rotenone-induced neurotoxicity and motor deficits is accompanied by a specific induction of the inflammatory molecule Eiger in both the young (1-day old) and the aged (20-days old) flies. We also observed an early induction of a JAK-STAT-mediated sterile inflammatory response. A few studies have implicated that the neurodegenerative molecule Sarm1 induced a proinflammatory cytokine response in viral encephalitis^24^. Interestingly, *dSarm* induction followed a similar pattern as *Eiger* expression in the rotenone-exposed flies and was induced early, reaching maxima at 20-days followed by its decrease. We also observed several fold higher induction of *dSarm* in the aged flies compared to the younger ones that corroborated our earlier result of age-associated increased susceptibility to rotenone. A recent study has suggested that Sarm1 deficiency allows mice with Nmnat2-deficiency related axonopathy to survive into old age without any phenotypic manifestations^33^. These observations strongly indicate that Sarm1 could be an important player in increased susceptibility to age-associated neuronal loss.

The reduction in *dSarm* levels after the initial peak following rotenone exposure could be due to the loss of neurons producing them per se in the rotenone-treated flies. In agreement with this, we demonstrated that short-term exposure to rotenone results in a progressive increase in locomotor deficits with accompanying *dSarm* induction indicating that an early induction of *dSarm* is necessary and sufficient for rotenone-induced locomotor deficits in flies.

To understand whether ROS plays an important role in rotenone-induced toxicity and accompanying Sarm1 induction and inflammatory response, we analyzed the ROS levels in the rotenone-treated flies. Our data indicated although there was a slight increase in the ROS levels following rotenone exposure in flies, there was no reversal in the survival or locomotor deficits in the flies when fed in media containing the ROS scavenger N-acetyl cysteine (NAC) in the presence of rotenone. This is in disagreement with some of the earlier studies where rotenone-induced ROS levels play an important role in regulating dopaminergic neuronal loss in mouse models of PD^34^.

To further understand the implication of rotenone-induced inflammatory response, flies were exposed to the naturally occurring polyphenolic compound resveratrol which has been shown to mediate anti-inflammatory responses in vitro. Since early induction of dSarm and the inflammatory responses were sufficient to induce the locomotor deficits and loss of survival in these flies, we conducted our studies in the younger flies (1-day following eclosion). Interestingly, resveratrol exposure not only reversed the activation of the inflammatory responses but also significantly reduced the expression of *dSarm* along with accompanying motor deficits thus indicating that dSarm may act downstream of the inflammatory responses in these flies. To pinpoint whether the inflammatory response acts upstream of Sarm1 induction, a time-point analysis was performed in the dopaminergic neuronal cell line SH-SY5Y where TNFα (human homolog of Eiger) preceded Sarm1 expression at 6 h post-treatment and was significantly reversed in the presence of Resveratrol. Importantly, using Sarm1 knockdown these cells.

**Fig. 8:**
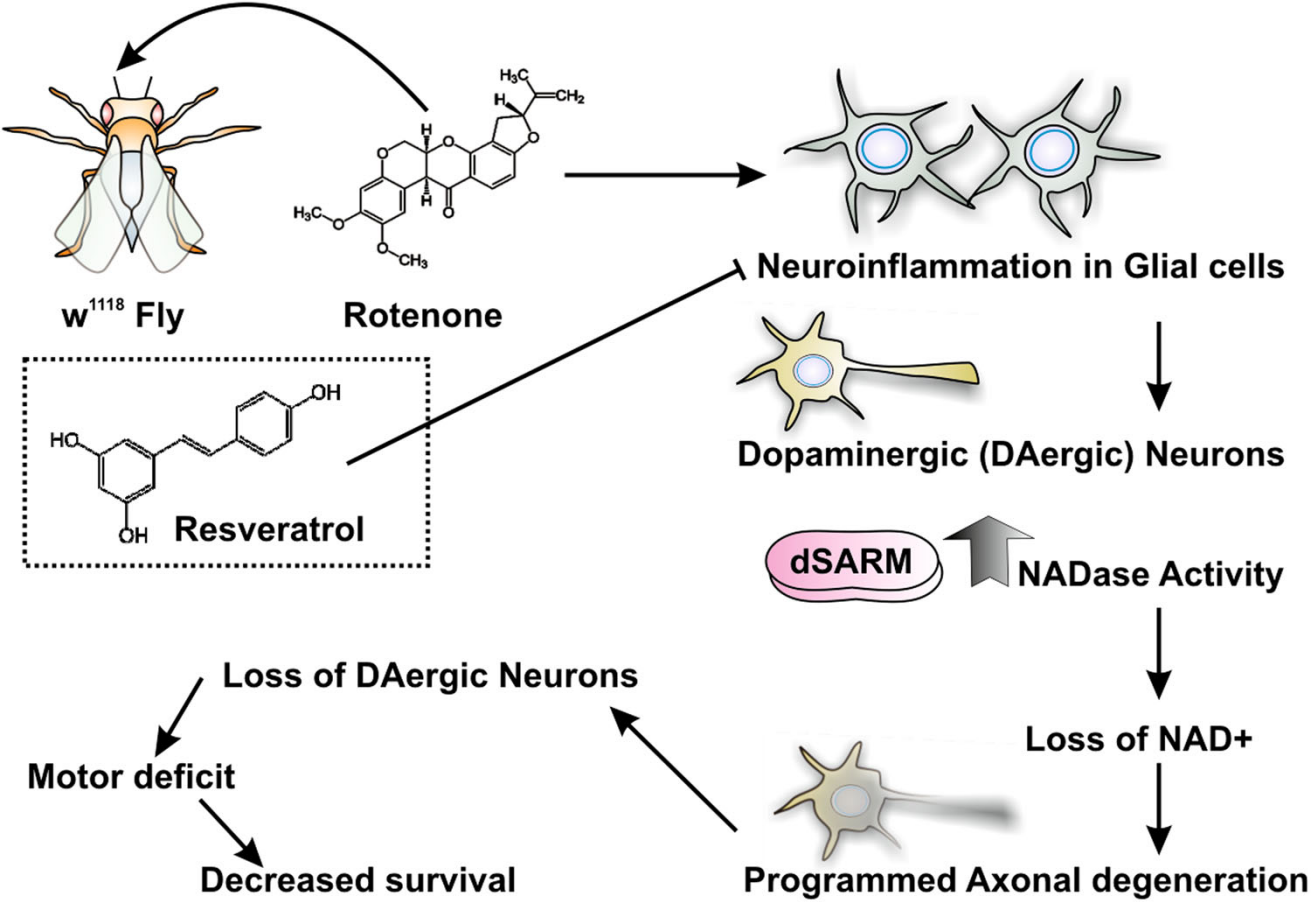
Proposed model for the study. Exposure to the pesticide rotenone stimulates Eiger production in the brain milieu of w^1118^ flies which further induces the increased expression of the NADase *Ect4* or *dSarm* from dopaminergic neurons. This, in turn, triggers the rapid loss of these neurons ultimately leading to severe motor deficits and loss of survival. Rotenone-induced neurotoxicity can be partially reversed in the presence of the anti-inflammatory molecule resveratrol which blocks the inflammatory response and subsequent *Ect4* induction leading to improved motor functions

In conclusion, our working model (Fig. 8) is based on the observation that rotenone treatment activates the inflammatory responses in the supporting cells of the CNS like microglia resulting in dSarm induction in the neurons. Activation of the dSarm signaling network results in rapid loss of dopaminergic neurons of w^1118^ flies that eventually leads to motor deficits and reduced lifespan in the rotenone-treated flies. This effect could be partially reversed in the presence of the anti-inflammatory molecule resveratrol indicating that targeting the inflammation induced dSarm pathway could play an important role in the age-associated neuronal loss. The aging model established in flies will help us better understand the increased susceptibility to age-dependent progressive loss of dopaminergic neurons following rotenone exposure. Thus targeting Sarm1 or the use of anti-inflammatory compounds to reduce its levels could open up cost-effective exciting avenues in the treatment of PD and other age-associated neurodegenerative diseases.

## Materials and methods

### Fly strains

Wild-type w^1118^ strains of fly were generously obtained from Dr. Rupak Datta, IISER, Kolkata. Both male and female flies were grown at 22 °C and 12-h light/dark cycle. Flies were fed on standard cornmeal/molasses diet and changed every 3–5 days. Only male flies were used in this study, since, female flies show altered physiological condition because of the reproductive development.

### Treatment

Cohorts of twenty flies of both genders per vials were placed on the Rotenone (Sigma, USA) containing diet at 1-day, 10-day, and 20-day post-eclosion. Rotenone was added to the fly food to achieve final concentrations of 50 , 100 , and 200 µM. Flies were transferred to fresh rotenone containing medium every 2 days and were monitored for survival and locomotor activity as described below. For aging studies, flies were grown for 20-days in regular medium following eclosion. At day-20 they were fed in rotenone containing diet and experiments were performed as described. For a few experiments flies were exposed to either rotenone or sodium arsenite (Nice Chemicals, India) in the presence or absence of 1 mg/ml of N-acetyl-L-cysteine (NAC) (Sigma, USA).

### Fly survival and negative geotaxis assay

Flies were chronically exposed to 50, 100, and 200 μM of rotenone 1-day, 10-day, and 20-days post-eclosion. For each condition, at least five vials containing twenty flies of both genders were raised on diet with or without Rotenone from 1-day post-eclosion and monitored daily for survival for up to 40-days. In order to determine the effect of rotenone exposure on locomotor ability negative geotaxis climbing assay was performed on the male flies fed on rotenone diet. Flies were tapped gently three times to the bottom of the vertical transparent tube (length, 25 cm; diameter, 3 cm) to initiate the negative geotaxis assay. After 10 s, the percentage of flies crossing the 5 cm mark was noted for each trial. A total of ten recordings were obtained per set and compared with untreated age-matched control flies. All behavioral experiments were performed at room temperature under standard light conditions.

### Resveratrol treatment

A dose-response curve was generated with varying conc. of resveratrol to monitor its effect on fly survival. Resveratrol was added to the fly food to achieve final concentration of 1 µM in the presence or absence of 200 µM rotenone. Flies were placed on rotenone containing food on the day of eclosion and transferred every other day to fresh vials and collected at 1 and 10-days post-treatment.

### Whole brain mount immunofluorescence labeling

The heads of adult flies were mechanically isolated under a dissection microscope and brains were dissected under stereozoom microscope (Olympus, Japan). Immediately after dissection brain were fixed in 4% PFA for 30 min. Brain tissues were incubated overnight in anti tyrosine hydroxylase (Merck Millipore) solution at 1:400 dilution in PBT (5% BSA and 0.3% Triton X-100 in PBS). Tissues were incubated with anti-rabbit Alexa 488 secondary antibody (Sigma, USA) for Tyrosine hydroxylase at 1:500 dilution in PBT for 3 h to detect primary antibody. After immunostaining tissues were mounted in vectashield (Vector Laboratories). Whole brain images were taken in Apotome 2 microscope (Zeiss, Germany), 9–12 tiles were taken in motorized stage and in each tiles images of 5 slices of 0.32 μm were taken and image were reconstructed by ZEN software.

### Isolation of RNA and qPCR

The heads of adult flies were mechanically isolated under a dissection microscope. The extraction of total RNA from fifteen male adult fly heads per sample was performed using Trizol (Invitrogen) reagent according to the manufacturer’s protocol. An additional centrifugation step was used at 10000 × *g* for 10 min to remove the cuticles prior to chloroform addition. 1 µg of total RNA were digested with DNAse I (NEB) followed by reverse transcription using the iScript Reverse Transcription Supermix (BIO-RAD) with oligo (dT) primers. Real-time PCR was performed on CFX96 Real-Time System (BIO-RAD) using the SYBR Green qPCR Master Mix (2 × ) from SsoFast EvaGreen Supermix (BIO-RAD) following the manufacturer’s instructions. Relative mRNA expression levels were normalized to glyceraldehyde 3-phosphate dehydrogenase (*Gapdh*) or *RPA70*. The quantification was performed using the comparative Ct method.

### ROS measurements

To assess the levels of ROS, 1-day old flies were grown in rotenone containing diet in the presence of absence of NAC. To measure ROS levels, heads of 10-day old flies treated as described were homogenized in extraction buffer containing 50 μM of 2,7-dichlorofluorescein diacetate (DCFDA) and incubated at room temperature for 60 min. Following incubation fluorescence was measured at 485 nm excitation/530 nm emission.

### Cell culture and treatments of SH-SY5Y cells

SH-SY5Y cells were obtained from the cell repository NCCS Pune, India and grown in DMEM-F12 (Gibco, Life Technologies) containing 10% v/v FBS (Gibco, Life Technologies) and 1% Penicillin streptomycin (Gibco, Life Technologies) at 37  °C and 5% CO_2_. For siRNA transfection assays, SHSY5Y cells were grown for 24 h and transfected with 25 nM of Sarm1 siRNA (Ambion, Life Technologies) (31 and 32), TNFα siRNA (Ambion, Life Technologies) or with a nontargeting siRNA (Ambion, Life Technologies) as a control using Lipofectamine 2000 (Invitrogen) for 24 h and following transfection cells were treated with 5 μM of rotenone for 24 h. For TNFα blocking studies, SH-SY5Y cells were cultured and pre-treated with TNFα neutralizing antibody (Abcam) at a final concentration of 5 mg/ml for 1 h to neutralize TNF α cytotoxicity activity in vitro. Cells were then treated with 5 μM of rotenone and cell survival was measured by MTT assay at 24 h post-treatment. RNA was also collected and RT-PCR was performed as described above to check the expression of SARM1 in these cells.

### MTT assay

3-(4,5-dimethylthiazol-2-ly)-2,5-diphenyltetrazolium bromide (MTT) was used to check cell viability. Cells were treated with rotenone in the presence or absence of TNFα blocker (TB) or siRNA against Sarm1 at the time points mentioned previously. Following treatment, cells were incubated with 0.5 mg/ml of MTT for 2 h at 37  °C and the absorbance was measured at 540 nm.

### Western blot analysis

Approximately twelve adult fly heads were homogenized per sample using a motorized pestle in lysis buffer containing 1 M NaCl, 1 M Tris, 0.5% Triton-X100 with protease inhibitor cocktail (Pierce Biotechnology) at 4 °C. After sonication, the lysates were cleared by centrifugation at 14,000 rpm for 15 minutes. Protein concentration was determined using Bradford assay kit (BioRad) and 15 μg of protein were loaded/well for subsequent western blot analysis using anti-Tyrosine Hydroxylase (Millipore) and β-Actin (Abcam) as loading control. For SH-SY5Y cells, cells were lysed using the M-PER protein extraction reagent (Thermo Fisher Scientific) containing a protease inhibitor cocktail (Pierce, Thermo Fisher Scientific). The levels of Sarm1 were analyzed using anti-Sarm1 antibody (Genetex) and β-Actin (Abcam) was used as a loading control.

### Statistical Analysis

Kaplan–Meyer survival analysis was performed using GraphPad Prism version 7.00 for Windows (GraphPad Software, La Jolla, CA, USA). The results are presented as mean ± SEM (standard error of mean) of at least three independent experiments performed in triplicate. Statistical significance was measured by one-way analysis of variance (ANOVA). Asterisks indicate levels of significance (*p < 0.05, **p < 0.01 and ***p < 0.001).

## Electronic supplementary material

Supplementary information

Fig. S1

Fig. S2

Table S1
