# Supplementary material for: Sarm1 induction and accompanying inflammatory response mediates age-dependent susceptibility to rotenone-induced neurotoxicity
Source: Cell Death Discov. 2018 Dec 11;4:114. doi: 10.1038/s41420-018-0119-5 (PMC6289984; doi:10.1038/s41420-018-0119-5)
Supplement: Supplementary file 1 — Supplementary information [file 41420_2018_119_MOESM1_ESM.docx]

**Supplementary Information**

**Table S1.** List of primers used in this study.

**Fig. S1. Exposure of w^1118^ flies to rotenone does not induce the expression of other immune response genes.** **(A)** Schematic representation of experimental setup for rotenone treatment in the young (1-day old) and the aged (10-day old) flies. **(B, C, D & E)** Total RNA was isolated from the heads of young and aged drosophila and exposed to 200 μM of rotenone for 10-days, converted to cDNA and subjected to real-time PCR with primer specific for *Upd1, Upd2, Upd3* and *Wnt 1* (n=5). *p ˂ 0.05, **p ˂ 0.01 and ***p ˂ 0.001 compared to control flies.

**Fig. S2.** **Exposure of w^1118^ flies to rotenone induce Sir2 gene expression but has no effect of Ampk expression. (A&B)** 1-day old flies were exposed to 200 μM of rotenone for 10 days in the presence or absence of resveratrol. *Sir2* and *Ampk* expression was measured suing real time PCR analysis. (n=3). *p ˂ 0.05, **p ˂ 0.01 and ***p ˂ 0.001 compared to control flies.
