# Supplementary figures and images for: Sarm1 induction and accompanying inflammatory response mediates age-dependent susceptibility to rotenone-induced neurotoxicity
Source: Cell Death Discov. 2018 Dec 11;4:114. doi: 10.1038/s41420-018-0119-5 (PMC6289984; doi:10.1038/s41420-018-0119-5)

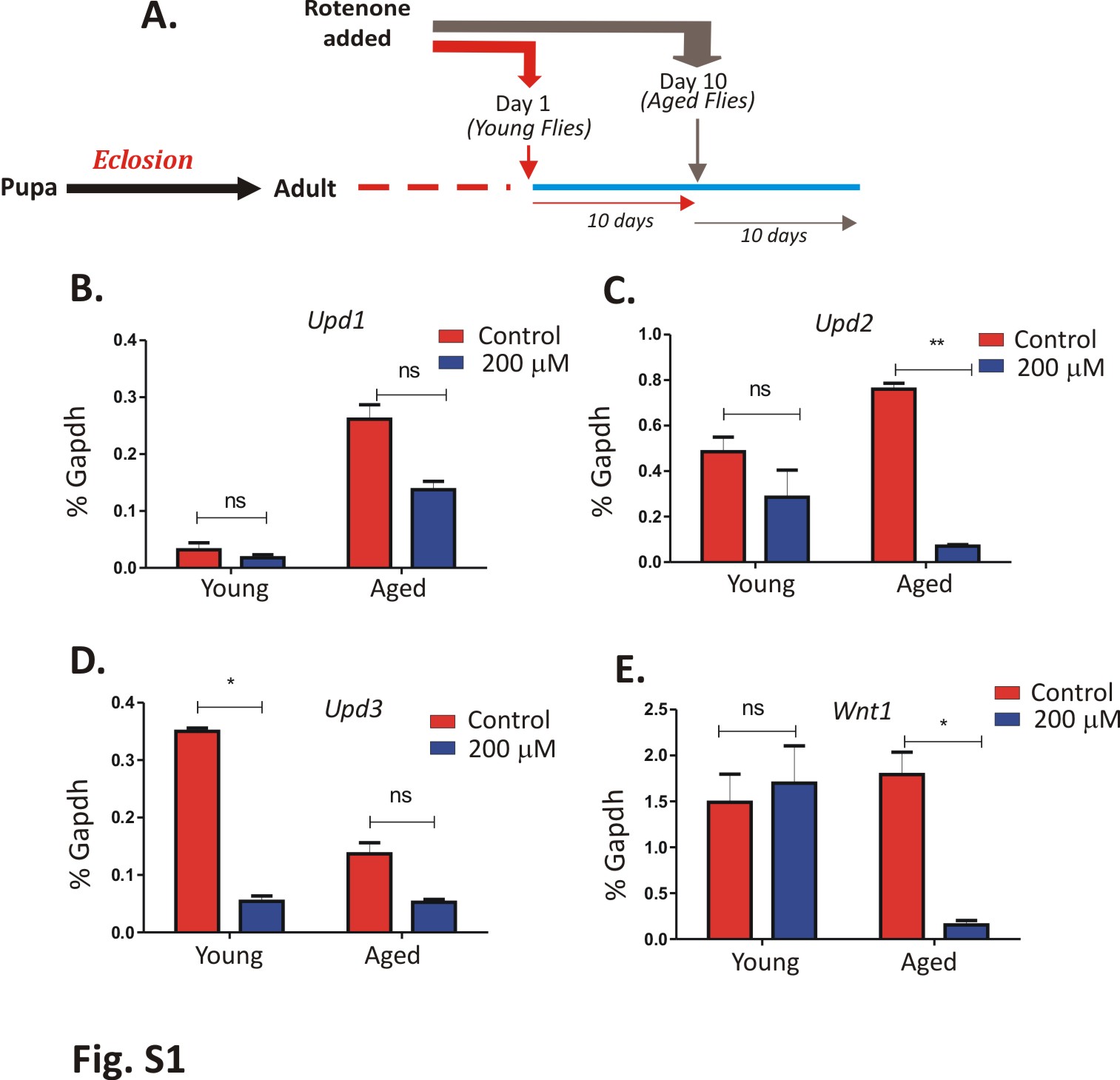

Supplement: Supplementary file 2 — Fig. S1 [file 41420_2018_119_MOESM2_ESM.jpg]

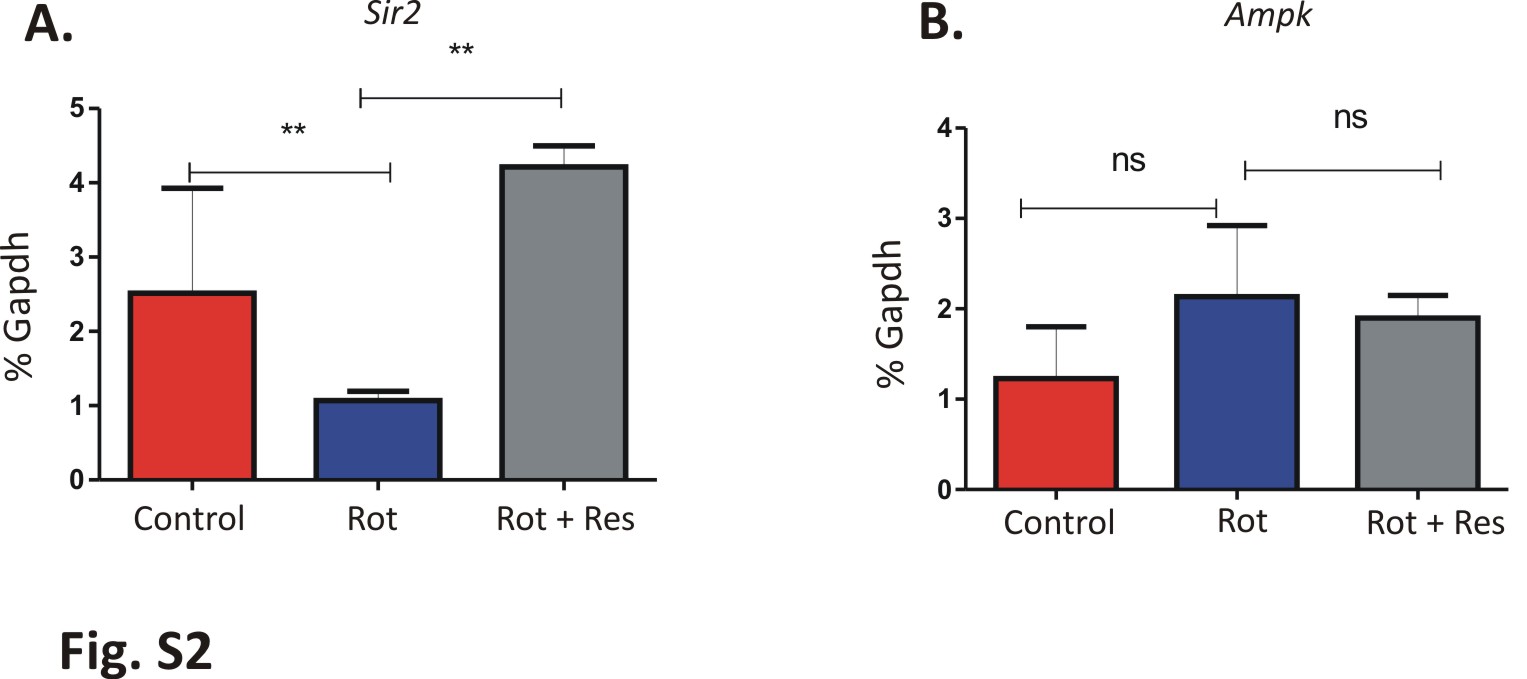

Supplement: Supplementary file 3 — Fig. S2 [file 41420_2018_119_MOESM3_ESM.jpg]
