# Supplementary material for: Sarm1 induction and accompanying inflammatory response mediates age-dependent susceptibility to rotenone-induced neurotoxicity
Source: Cell Death Discov. 2018 Dec 11;4:114. doi: 10.1038/s41420-018-0119-5 (PMC6289984; doi:10.1038/s41420-018-0119-5)
Supplement: Supplementary file 4 — Table S1 [file 41420_2018_119_MOESM4_ESM.docx]

*Table S1 : The primer sequences used in this study are as follows:*

| **Gene** | **Forward primer** | **Reverse primer** |
| --- | --- | --- |
| *Gapdh* | GTGGCCGTCAACGATCCCTTC | TTGATGTTGGCCGGGTCGC |
| *RP49* | GACGCTTCAAGGGACAGTATCTG | AAACGCGGTTCTGCATGAG |
| *Ect4* | AAGGAAGCCCTCTCACTCCGC | ACTTTGCACCGTAATACTGGAGGCC |
| *Eiger* | GACTGCCGAGACCCTCAAGC | TGGTCGCTTTGGCCGGAAAA |
| *TotM* | GCCAAGCCTGCACTATGAAT | GCTGTCGATGTTCCGGTATT |
| *Tep1* | TCTGTAAAGCGGGGTGAAGT | CAGAGTAGTCAGGCCCACTT |
| *Tep2* | CCTCATGGGTGGTTACTGGT | AGCAATTACCTCACCTCGCT |
| *Upd1* | GCATCGAAGCACCCGACC | AACTGCTATCACTGTTCCTCCCTGA |
| *Upd2* | GCCAGCCAACAGAGCCAAA | ATCACTAGCAGCACCTGCCG |
| *Upd3* | TTCGTCCAGCCGCGATATAAAGATAC | CAG GCG AAT CAG GCG ACT TTG |
| *Wnt* | TATTACGAGCACTCTGGCAGCAG | AGGAGAGTGGTGCCTGGAAC |
| *Sir2* | ACATACACACACAGGCGCAGCTA | TTCGTAATTTTCGAGCGTAGTTTCTGGAA |
| *Ampk* | GCCGCGCTCGAATAGAAATCCA | GATGTCAAAGTGGTTGTGGATGTGTC |
| *Human Gapdh* | CCCATCACCATCTTCCAGGAGC | GCCTTCTCCATGGTGGTGAAGAC |
| *Human SARM1* | GAGCAGATCCTGGTGGCTGAGAA | GCTTGAACATGTGCTCCAAGATGCC |
| *Human TNFa* | GAGTGACAAGCCTGTAGCCCA | GGTGTGGGTGAGGAGCAC |
